# Disseminated Penicillin-Resistant Streptococcus pneumoniae Infection: A Case Report

**DOI:** 10.7759/cureus.59225

**Published:** 2024-04-28

**Authors:** Ken Kodama, Toru Momozane, Hiroshi Takehara, Masanori Kaneko, Hirotsugu Honda

**Affiliations:** 1 Department of Thoracic Surgery, Yao Municipal Hospital, Yao, JPN; 2 Department of Orthopedic Surgery, Yao Municipal Hospital, Yao, JPN

**Keywords:** odontogenic maxillary sinusitis, lumbar spondylodiscitis, sternoclavicular joint septic arthritis, sepsis, penicillin-resistant streptococcus pneumoniae (prsp)

## Abstract

An invasive pneumococcal disease involving sternoclavicular joint arthritis, lumbar spondylodiscitis, and muscular abscesses caused by penicillin-resistant *Streptococcus pneumoniae* has not been reported previously. We successfully treated a 57-year-old man with this condition using surgical drainage and debridement, and laminectomy/fenestration, in combination with the administration of two IV antimicrobial drugs based on blood culture results. Clinical resolution was obtained after decompression of the lumbar spine, with minimal restriction of the left lower limb. This treatment approach should be considered depending on the pathogen, underlying host factors, and the severity of the disease.

## Introduction

Sternoclavicular joint (SCJ) septic arthritis accounts for approximately 1% of all joint infections [1]. Spondylodiscitis is the main manifestation of hematogenous osteomyelitis in patients over 50 years old and represents 3-5% of all osteomyelitis cases [2]. A common feature of these illnesses is that *Staphylococcus aureus* (*S. aureus*) is the causative bacterium, responsible for 49% of SCJ septic arthritis cases and 20-80% of spondylodiscitis cases, with intravenous drug users being more commonly affected [1, 2]. As this type of arthritis may be part of the systemic symptoms of sepsis, these infections hold clinical significance for physicians across all specialties, particularly those in primary care, emergency medicine, infectious disease, thoracic surgery, and orthopedic surgery. Due to the ambiguity of presentation and low prevalence, diagnosis and treatment are often delayed.

However, *Streptococcus pneumoniae* (*S. pneumoniae*) is a rare cause of septic arthritis, responsible for 2-4% of cases, with a higher prevalence noted in some cohorts [3]. Herein, we report a successfully treated case involving an adult male with SCJ septic arthritis, lumbar spondylodiscitis, and multiple abscesses (bilateral psoas muscle, right serratus anterior, left trapezius muscle, and thyroid) caused by penicillin-resistant *S. pneumoniae* (PRSP).

## Case presentation

**Figure 1:**
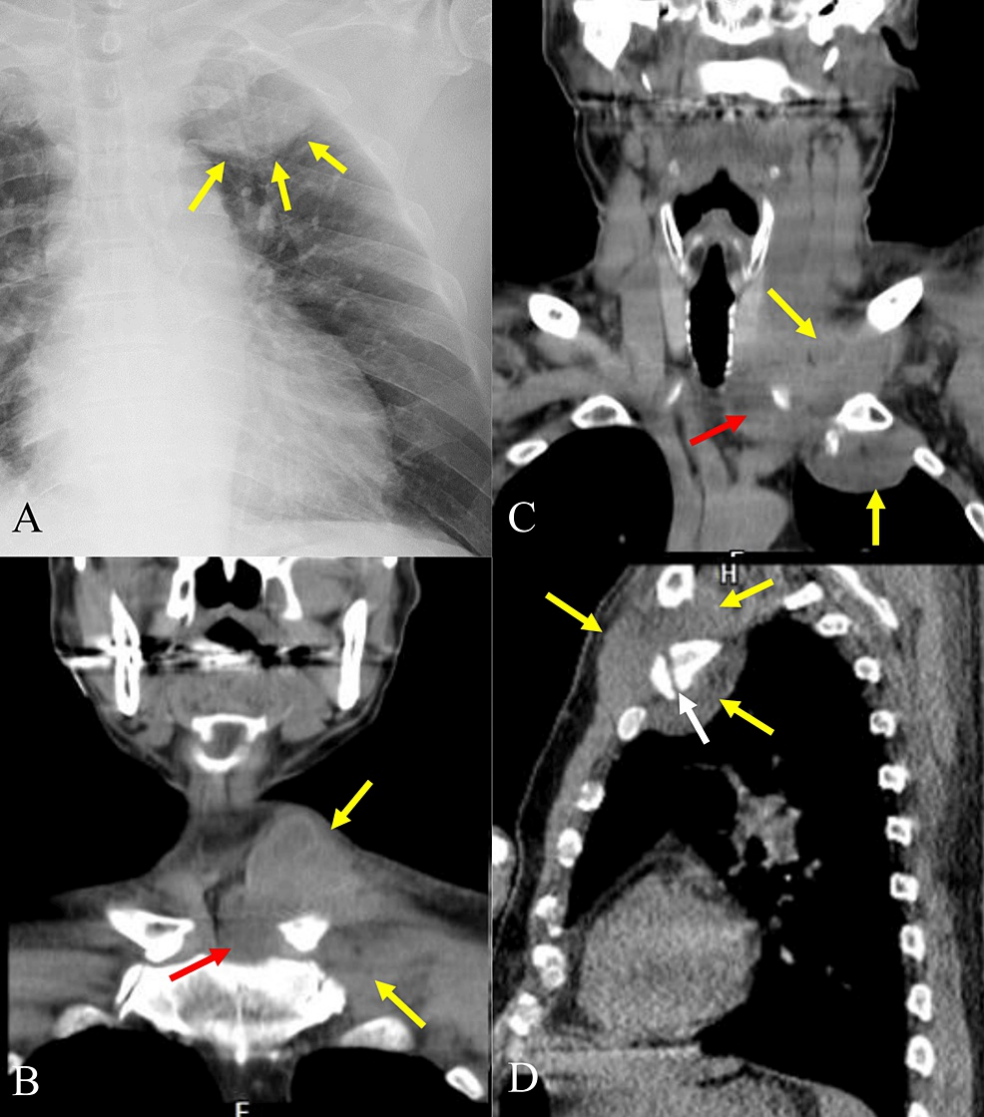
Initial chest roentgenogram and computed tomography. A: Initial chest roentgenogram showing homogeneous density in the left chest apex (yellow arrows). B-D: Computed tomography showing a periarticular abscess surrounding the left sternoclavicular joint (yellow arrows). There is abnormal fluid density along the medial aspect of the clavicular head (red arrows), but bone erosion and cortical irregularity were not noted. Widening of the costochondral junction of the 1st rib was demonstrated (white arrow).

On hospital day 1, a 57-year-old man was referred to our hospital with a 2-week history of progressive bilateral shoulder pain, neck and lower back pain, gait disturbance, and fever. A chest roentgenogram revealed an abnormal shadow in the left pleural apex. During the physical examination, he complained of marked redness and tender swelling around both shoulders and the left SCJ. He had difficulty walking and reported lower back pain. At presentation, his vital signs were as follows: blood pressure = 113/75 mm Hg; heart rate = 89 beats/min; oxygen saturation = 95% in room air; temperature = 37.8°C. Laboratory investigations revealed leukocytosis of 29,800 WBC/mm3 (normal range: 3,900-9,800). His C-reactive protein (CRP) level was 29.64 mg/dL (normal range: 0-0.14 mg/dL). His past medical history included clipping surgery for a cerebral aneurysm, and due to the unknown material of the clip, magnetic resonance imaging (MRI) was not employed for this patient. He denied any history of trauma or other risk factors such as diabetes or drug abuse. However, according to his interview, he had a recent history of painful swelling of the right upper gingiva, which was treated by removing a dental bridge. He was a current smoker with a 77 pack-year history. A chest roentgenogram revealed a 6.6 x 6.5-cm well-defined homogeneous mass shadow at the apex of the left chest (Figure 1A). Neck and chest computed tomography (CT) without contrast revealed a periarticular abscess around the left sternoclavicular joint (Figures 1B-1D). There was abnormal fluid density along the medial aspect of the clavicular head, but no bone erosion or cortical irregularity was noted (Figures 1B, 1C). Widening of the costochondral junction of the 1st rib was demonstrated (Figure 1D).

On the same day, emergent surgical debridement was performed by a surgical team comprising both thoracic and orthopedic surgeons. The left SCJ was approached through a parallel 6-cm cervical hemi-collar incision and an incision at the left 2nd rib. Abundant purulent material was evacuated from both the periarticular abscesses and articular space. Macroscopically, the bone structures in the sternoclavicular joint were intact. Bilateral deltopectoral approaches were used to evacuate and debride the abscesses extending from the coracoid process to the clavicula and posterior to the acromion of the right shoulder, and the abscess extending from the coracoid process to the upper part of the clavicula and the back of the scapula of the left shoulder. After extensive irrigation and debridement of all necrotic tissue, two closed-suction drains were placed deep in the pectoralis and retrosternal spaces and one in each of the abscess cavities around both shoulder joints. Postoperative empirical antibiotic treatment was started intravenously with meropenem (MEPM) 1,000 mg three times a day and vancomycin (VCM) 1,000 mg twice a day.

Penicillin-resistant *S. pneumoniae* (PRSP) was isolated from peripheral blood, purulent material evacuated from all three abscesses, and sputum. The serotype of PRSP was 23F. Antimicrobial susceptibility tests using these cultures showed the following results: susceptible to amoxicillin/clavulanate (AMPC/CVA): minimum inhibitory concentration (MIC, μg/mL) = 2, clindamycin (CLDM): MIC ≤ 0.03, tetracycline (TC): MIC ≤ 0.5, chloramphenicol (CP): MIC ≤ 2, VCM: MIC = 0.5, levofloxacin (LVFX): MIC = 1, gatifloxacin (GFLX): MIC = 0.5, and sulfamethoxazole/trimethoprim (ST): MIC ≤ 10; intermediate to cefotaxime (CTX): MIC = 2, ceftriaxone (CTRX): MIC = 2, cefepime (CFPM): MIC = 2, and imipenem/cilastatin (IPM/CS): MIC = 0.5; resistant to penicillin G (PCG): MIC > 8, cefuroxime (CXM): MIC > 4, MEPM: MIC = 1, and erythromycin (EM): MIC > 1. The PCG criteria for *S. pneumoniae* were meningitis: S ≤ 0.06, R > 0.12, and for cases other than meningitis: S ≤ 2, I = 4, R > 8.

**Figure 2:**
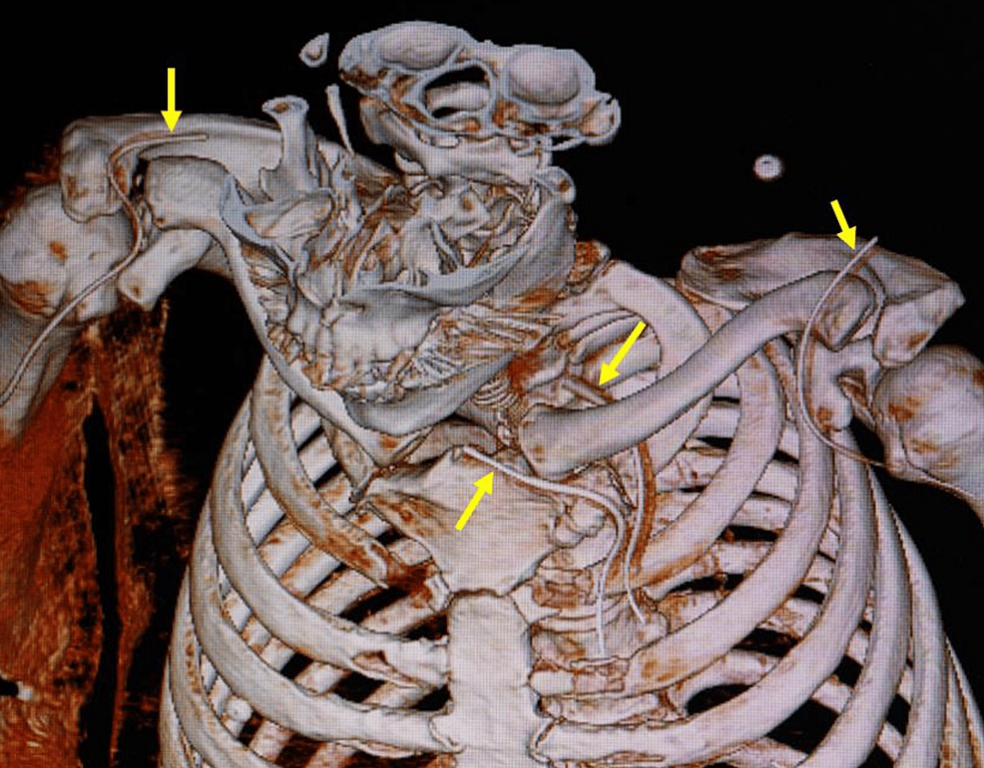
Post-operative volume-rendering image of the chest and shoulders. Four catheters (indicated by arrows) placed in the abscesses above and below the left sternoclavicular joint, and in the abscesses around the deltoid muscles of both shoulders.

On hospital day 2, a whole-body CT with contrast was conducted, revealing that all four closed-suction drain tubes were placed in the abscess cavities around both shoulder joints and in the anterior and posterior abscess cavities around the left SCJ (Figure 2).

**Figure 3:**
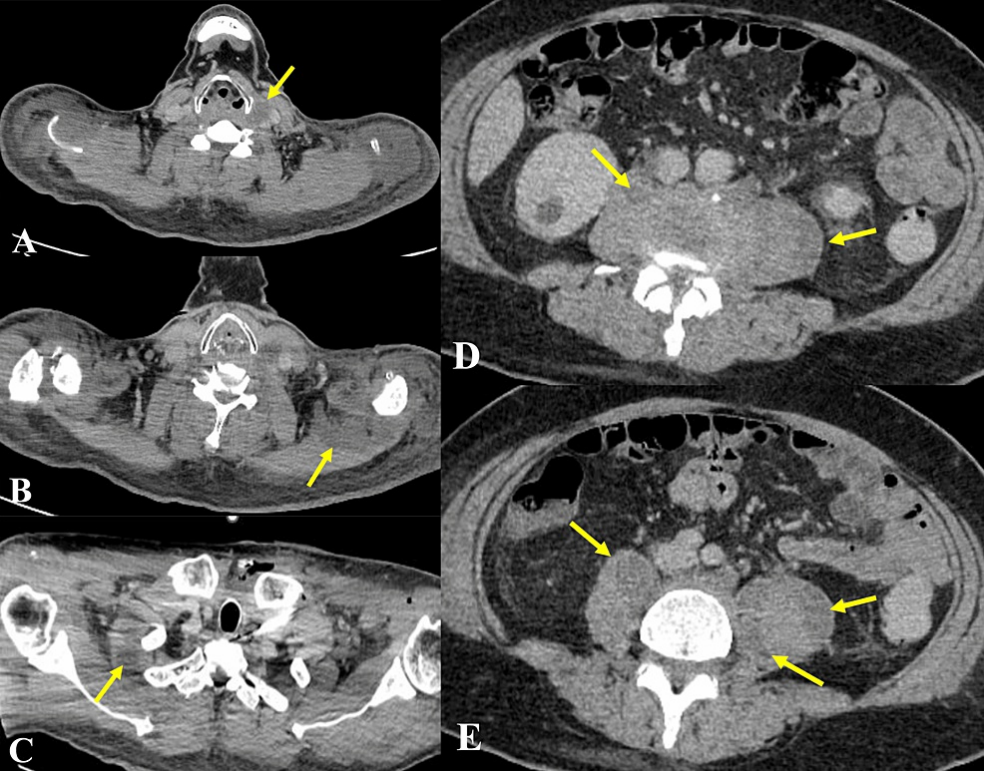
CT taken on hospitalization. Multiple abscesses located on the left side of the thyroid gland (A), ventral to the left trapezius (B), in the right serratus anterior (C), and in the bilateral psoas majors (D, E).

On that CT, we identified multiple abscesses located in the bilateral psoas majors, the right serratus anterior, on the left side of the thyroid gland, and ventral to the left trapezius (Figure 3).

On hospital day 4, a dental examination revealed poor oral health care, severe but asymptomatic periodontitis in the upper right wisdom tooth, and maxillary sinusitis. Transthoracic cardiac echocardiography showed no vegetation or structural abnormalities of the valves. On hospital day 5, the blood culture turned negative.

**Figure 4:**
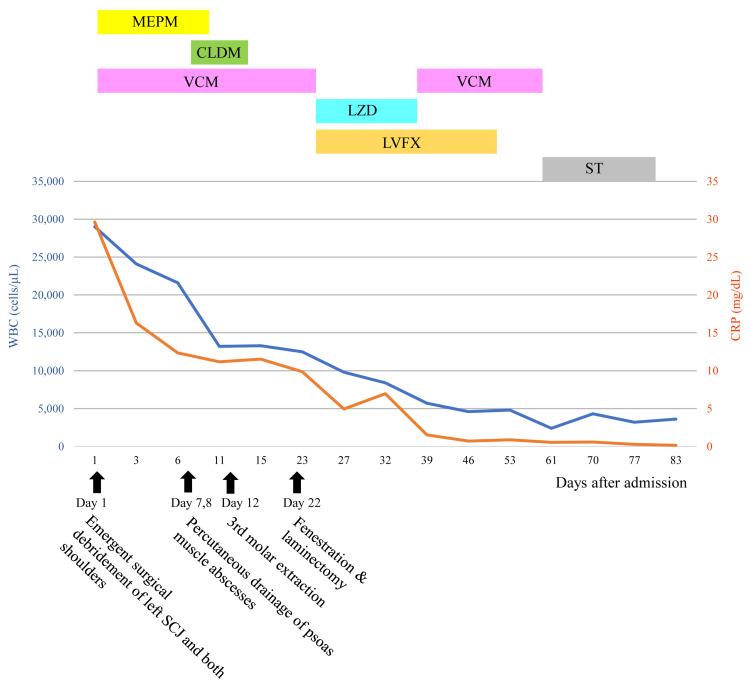
Clinical course. MEPM: Meropenem; CLDM: Clindamycin; VCM: Vancomycin; LZD: Linezolid; LVFX: Levofloxacin; ST: Sulfamethoxazole/Trimethoprim combination; SCJ: Sternoclavicular joint.

On hospital days 7 and 8, percutaneous drainage of the two abscesses located in the left psoas muscle was performed under CT fluoroscopic guidance. A 6-French drainage catheter was accurately inserted into each abscess cavity and left in place for 6 days. Based on the results of an antimicrobial susceptibility test, MEPM was de-escalated to CLDM 600 mg three times a day until hospital day 12, and vancomycin was continued at increasing doses up to 1,750 mg twice a day until hospital day 23 (Figure 4).

On hospital day 12, the upper right third molar was extracted due to severe odontogenic sinusitis.

On hospital day 19, the patient showed impaired awareness and became hypoactive. A brain CT was performed to check for a brain abscess, but no mass lesion was detected. Streptococcal meningitis was also suspected; however, bacterial testing of spinal fluid was negative, and the neuropsychiatric symptoms disappeared along with an improvement in the CRP value.

**Figure 5:**
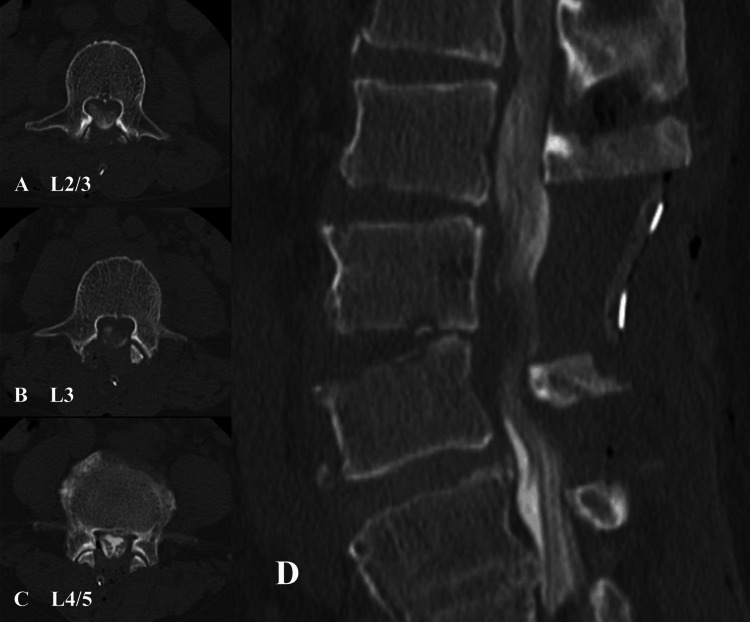
Pyogenic spondylitis. Postoperative CT myelography. Fenestration of L2/3 (A) and L4/5 (C), and laminectomy of L3 (B) were performed. The joint capsule and articular processes of the L3/4 facet joint were destroyed and protruded; they were removed due to necrosis. A purulent substance was evacuated from the dorsal side of the epidural space, and the hypertrophied yellow ligament was resected. After debridement, compression on the dural sac was completely removed (D). The posterior longitudinal ligament was intact, so it was preserved to support spinal stabilization.

On hospital day 22, although the white blood cell count, CRP level, and body temperature were steadily decreasing, the skin distal to the level of the umbilicus showed progressive hypoesthesia. Lower limb paresis and back pain were also progressive, and physical findings revealed bilateral weakness in the quadriceps femoris and tibialis anterior muscles. Based on these findings, we strongly suspected progressive lumbar spondylodiscitis. Therefore, fenestration of L2/3 and L4/5 and laminectomy of L3 were performed. The joint capsule and articular processes of the L3/4 facet joint had been destroyed and protruded due to osteomyelitis. An abundant purulent substance was evacuated from the dorsal side of the epidural space, and the hypertrophied yellow ligament was resected. After debridement, thecal sac neuro-compression was completely released. The posterior longitudinal ligament was intact, so it was preserved to support spinal stabilization. Spine fusion surgery was not performed. After extensive irrigation, a 5.0 mm diameter closed-suction drain was placed in the epidural space. Postoperative CT myelography is shown in Figure 5.

On hospital day 24, because infection control was deemed insufficient based on findings during lumbar spine surgery, a switch was made to linezolid (LZD), an antibacterial agent with better tissue penetration to bone than vancomycin. Thus, LZD 600 mg twice a day was administered until hospital day 35. In addition, oral LVFX 500 mg once a day was administered to prevent meningitis until hospital day 51.

On hospital day 36, VCM 1,000 mg twice a day was restarted and 1,750 mg twice a day was administered from hospital day 37 to day 60, followed by oral administration of ST (400mg/80mg) combination of 2 tablets twice daily until hospital day 81 (Figure 4).

On hospital day 89, he was discharged.

Since hospital day 46, the CRP value has remained lower than 1.00 mg/dL. Based on a 12-month follow-up, his general condition remains good with no recurrence of infection. There are no restrictions on his upper body functions. Although he still has some difficulty in flexing his left lower limb, he can walk for approximately 3 km. Also, there is no functional impairment of the right lower limb.

## Discussion

SCJ septic arthritis remains an unusual clinical problem. Several risk factors have been associated with SCJ infection, including intravenous drug use, concomitant infection at a different location, diabetes mellitus, trauma, and central venous line-related infection. Only 23% of patients have no identifiable risk factor [4]. Asymptomatic odontogenic sinusitis with poor oral health care and heavy smoking may also be included in this patient group. The present case had odontogenic maxillary sinusitis and involved active gingivitis before the onset of the septic symptoms, which is considered to be the original site of bacteremia. In this case, abscesses formed throughout the body as a result of systemic bacteremia, and the mechanism is assumed to be that *S. pneumoniae* was transmitted to the SCJ and lumbar discs either through these abscesses or directly from the systemic bacteremia. A localized infection which progresses into an uncontrolled systemic response is usually the cause of sepsis.

Management of patients with SCJ septic arthritis consists of surgical debridement and antibiotics. Conservative treatment alone is frequently ineffective since extra-articular complications are usually present at the time of diagnosis [5]. Because of a high recurrence rate and persistent latent infection reported in some studies, some authors have proposed a more aggressive approach, with formal joint resection, including the medial clavicle, lateral sternal manubrium, and first and second ribs, especially in cases of osteomyelitis. If necessary, pectoralis muscle coverage should be considered [5-7]. A single-stage resection and muscle advancement flap lead to a higher incidence of complications. Debridement with open wound care provides satisfactory outcomes with minimal perioperative complications but requires prolonged wound care, as in our patient [8]. Recently, Schreiner W et al. [9] reported that negative pressure wound therapy combined with instillation and dwell time may be a useful strategy in patients with SCJ septic arthritis, leading to a higher incidence of bacterial eradication and shorter wound care.

Although early diagnosis and treatment are desirable, abscesses have often already formed throughout the body by bacteremic seeding [4], as in our case. Therefore, abscesses requiring surgical intervention must be carefully selected based on the severity of local symptoms and the administration of effective antibiotics with proven susceptibility. Fortunately, the macroscopic bone structures in the sternoclavicular joint were intact, so surgical drainage was performed without joint resection in our case. Instead, surgical drainage around both shoulder joints was simultaneously performed. Subsequently, the pathogenic bacterium was proven to be *S. pneumoniae*, and the findings in the oral cavity suggested that the sepsis was due to odontogenic maxillary sinusitis, so the affected tooth was extracted to control the chronic infection at this site, and the administration of antibiotics based on drug susceptibility was continued. Nevertheless, the symptoms of lumbar spondylodiscitis worsened, forcing us to perform laminectomy/fenestration before the patient became completely paralyzed.

It is unclear whether this lumbar spondylodiscitis can spread hematogenously or from direct extension to or from contiguous structures, such as the psoas muscle [10]. Fortunately, the patient made a smooth recovery after decompression without additional spinal fusion surgery. He is now able to walk and drive a car, although the range of motion in his left hip joint is limited. We consider this sequela a result of damage to the lumbar nerve plexus due to left psoas abscesses. Additionally, after continuing antibiotics for 81 days, there was no recurrence of infection, and he is now leading a normal daily life. According to the literature, the duration of antimicrobial therapy for spinal infection ranges from 6 to 12 weeks [2]. Positive blood cultures, neurological abnormalities, and staphylococcal infections are associated with longer intravenous courses. However, there is no data specifically for spondylodiscitis with PRSP. Recrudescence of infection is known to occur even years after spondylodiscitis has been treated [2]. Therefore, long-term follow-up after surgery is necessary.

Our patient with PRSP was successfully treated with a combination of VCM and the sequential use of VCM to LZD and LZD back to VCM, based on the results of antibiotic susceptibility testing. Pneumococcal septic arthritis is usually associated with a favorable prognosis when appropriate treatment is instituted rapidly. Most patients recover and achieve their initial joint range of motion or develop only minor sequelae with a mildly reduced range of motion, as in our case. There is no consensus regarding the duration of intravenous therapy (usually 3-4 weeks, but 6-8 weeks for spinal infections) or the role of subsequent oral therapy [11].

Severe pneumococcal disease may be prevented by pneumococcal vaccines [12]. Guidelines currently recommend pneumococcal vaccination among patients at risk of severe disease, identifying age and comorbidity as the main risk factors [12]. For healthy people, the target population for pneumococcal vaccination under the Japanese national immunization program includes adults aged 65 years and older [13]. The present case involved a 57-year-old man considered a healthy adult because his *S. pneumoniae* odontogenic infection was asymptomatic, and therefore, he did not receive the pneumococcal vaccine. In the future, it will be necessary to consider whether guidelines cater to decision-making for pneumococcal vaccination in individual patients with *S. pneumoniae* isolated from asymptomatic lesions.

## Conclusions

We presented a case of a patient with multiple abscesses and left sternoclavicular arthritis, along with progressive lumbar spondylodiscitis due to PRSP sepsis, who was successfully treated with surgical drainage and debridement for left sternoclavicular arthritis and abscesses around the deltoid muscles of both shoulders, and laminectomy/fenestration of lumbar vertebrae II-IV. The treatment was administered under two intravenous antimicrobial drugs. To date, he has shown no signs of recurrence for 12 months. The treatment should be considered depending on the pathogen, underlying host factors, and the severity of the disease.
